# Supplementary figures and images for: Video abstracts and plain language summaries are more effective than graphical abstracts and published abstracts
Source: PLoS One. 2019 Nov 19;14(11):e0224697. doi: 10.1371/journal.pone.0224697 (PMC6863540; doi:10.1371/journal.pone.0224697)

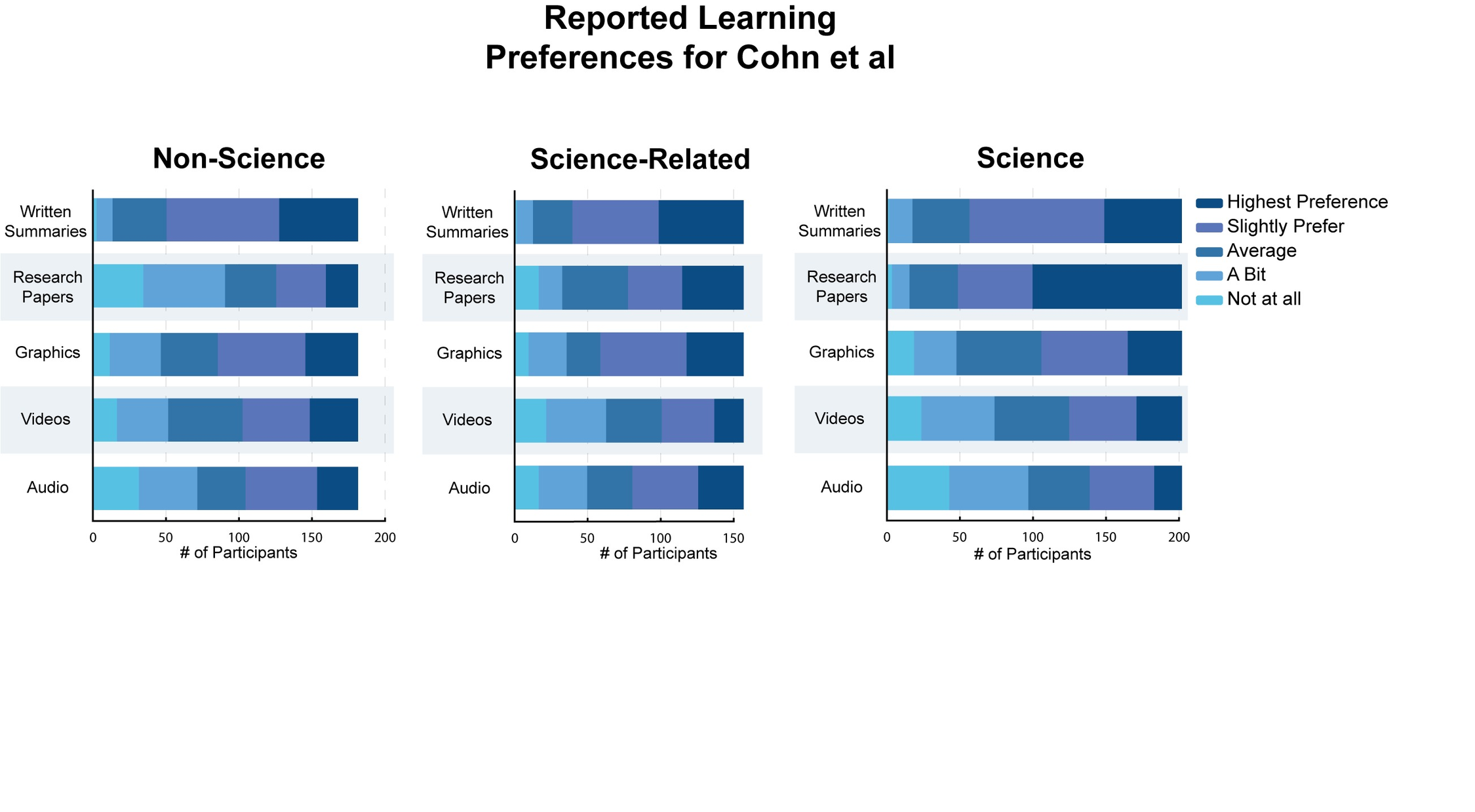

Supplement: S1 File — The bar charts show the reported preference of the participants for different ways to hear about science separated by career for the Cohn et al. data set. (TIF) [file pone.0224697.s001.tif]

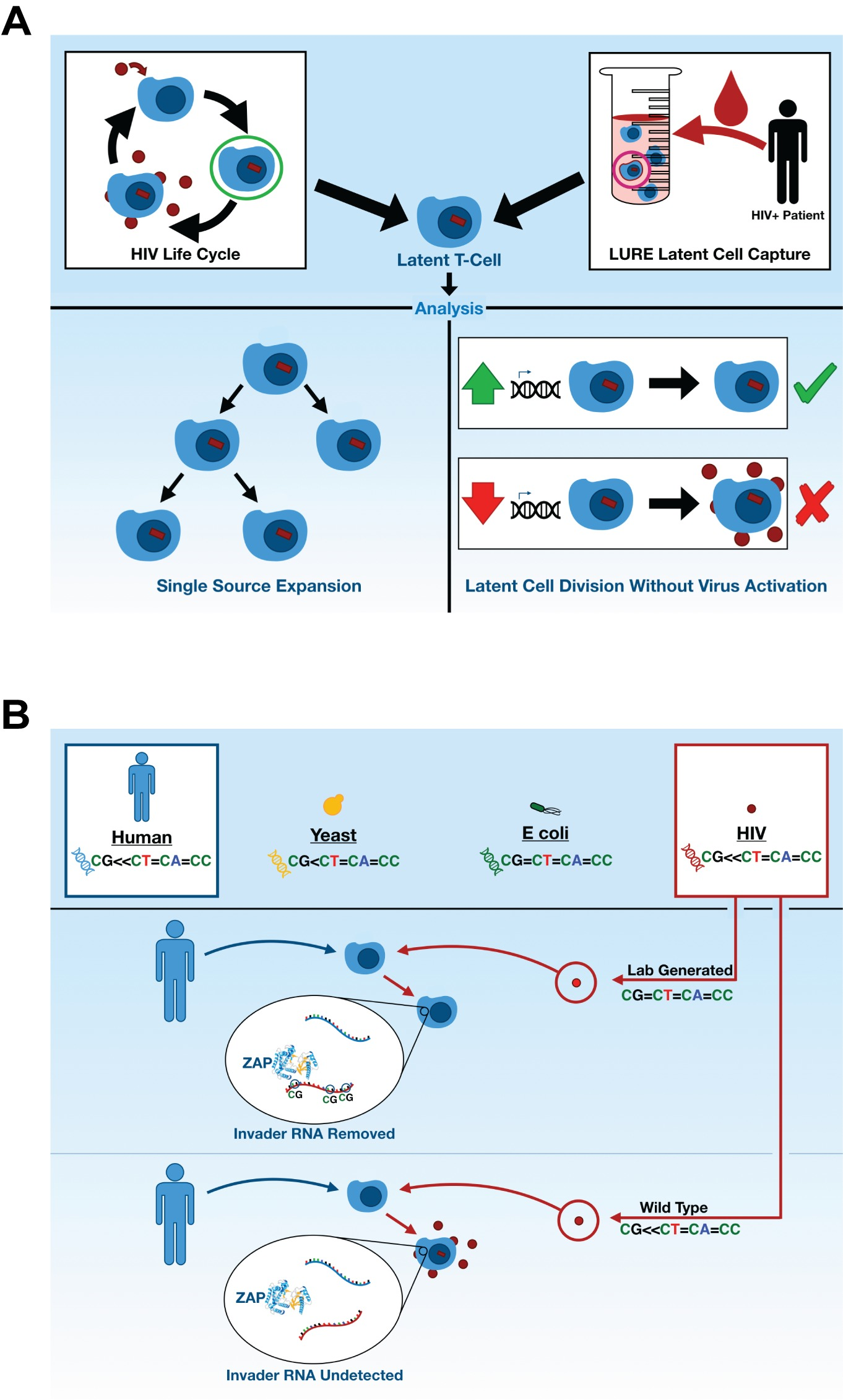

Supplement: S4 File — Graphical abstracts created for the Cohn et al. (A) and Takata et al. (B) papers. Graphical abstracts used similar visual motifs as the video abstracts and were created using Keynote software. Each abstract was put through a color blindness simulator to ensure that the abstracts could be seen properly by all viewers. The abstracts were embedded into the survey for participants to review. (TIF) [file pone.0224697.s004.tif]

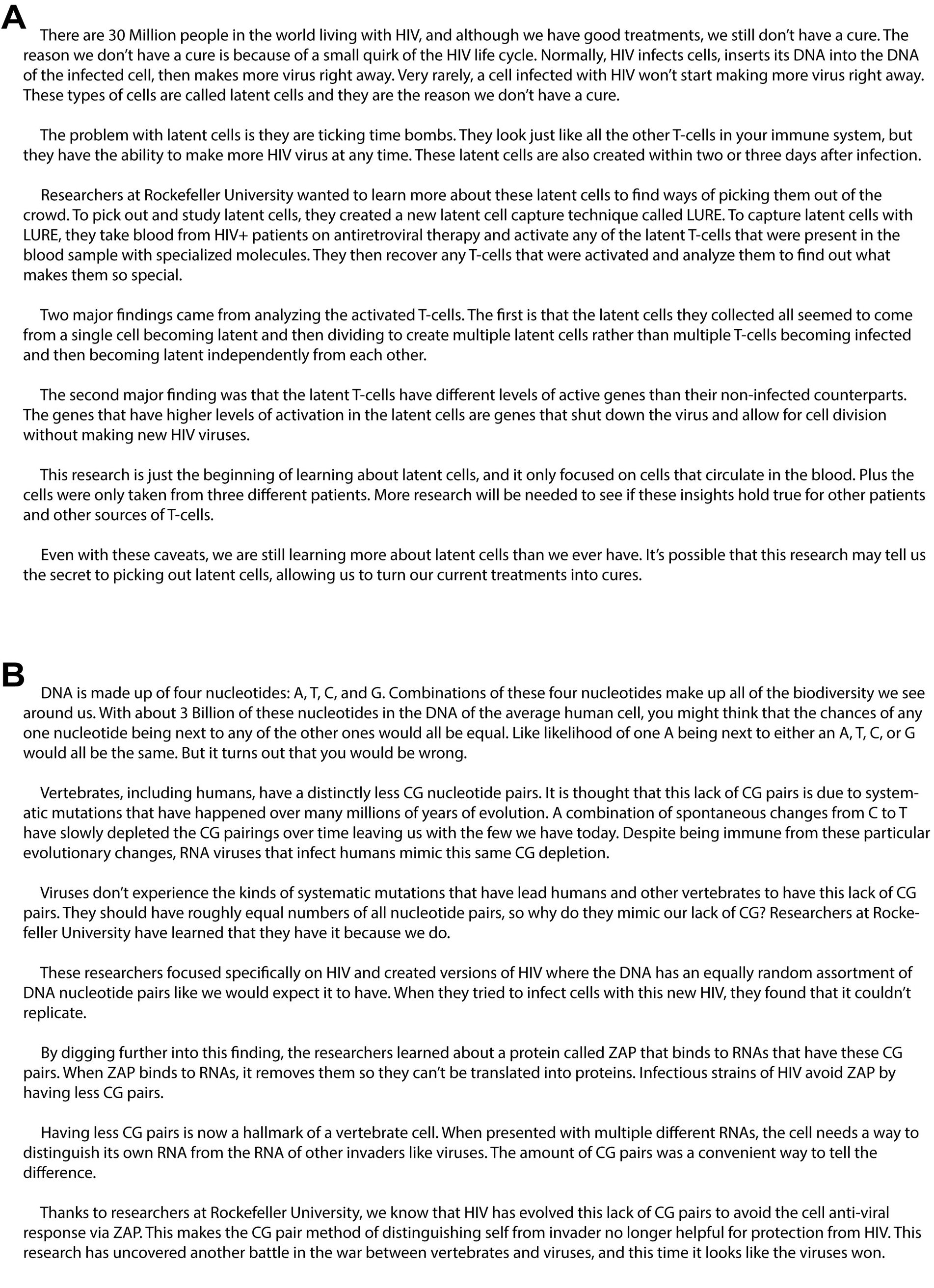

Supplement: S5 File — Plain language summaries written for the Cohn et al. (A) and Takata et al. (B) papers. Summaries were written based on intensive review of the published papers. The summaries also hit each key point mentioned in the abstracts of each paper. The Cohn et al. summary contains 422 words (A) and the Takata et al. summary contains 433 words (B). Each summary was embedded into the survey for participants to review. (TIF) [file pone.0224697.s005.tif]
