# Supplementary material for: Video abstracts and plain language summaries are more effective than graphical abstracts and published abstracts
Source: PLoS One. 2019 Nov 19;14(11):e0224697. doi: 10.1371/journal.pone.0224697 (PMC6863540; doi:10.1371/journal.pone.0224697)
Supplement: S6 File — A PDF copy of the survey presented to participants. This copy shows the published abstracts as the summary type. It has the Cohn et al. summary shown first and the Takata et al. shown second. Other surveys are identical to this one except that they show videos, plain language summaries, or graphical abstracts instead of the published abstracts. The videos can be seen in S2 and S3 Files. The graphical abstracts are in S4 File and the plain language summaries are in S5 File. Half of the surveys have the Cohn et al. summary shown first and half have the Takata et al. summary shown first. (See Fig 1 for schematic). (PDF) [file pone.0224697.s006.pdf]

Accessibility of Research Papers

Principal Investigator: Kate Bredbenner
Phone: (212) 327-8150
E-Mail: kbredbenne@rockefeller.edu

Title of the research study: Investigation of research paper accessibility methods regarding different science-interested populations

• You are being asked to participate in this survey because we are generating data about the comprehension and enjoyment of a variety of scientific summary presentations.

• You will be asked questions about your previous educational background and your learning preferences. You will also be shown a scientific presentation and be asked comprehension questions.

• The survey should take no longer than 25 minutes to complete.

• This study will provide no direct benefit to you.

• Your participation in this survey is entirely voluntary.

• You will be assigned a code and a timestamp for the study, and your answers will be linked to that code and timestamp only.

• The survey will be presented using the online survey application Google Forms. Data you input will be sent and stored on a central server that is protected by high-end firewall systems and on which vulnerability scans are performed regularly.

General Information for participating in any online activity:

• Just as with any information that is entered online, there is a small chance that confidential information could be made public. As researchers, we use very high level security systems to make sure that this chance is small.

• For this study the Google platform will have access to your IP address and your survey response. Google will know your small text files (cookies) that you previously accessed which may result in your receiving unsolicited emails.

• Third party tracking and sale of data, preferences, survey accessing, etc., may result in presentation of other surveys.

By answering the questions in this survey, you are consenting to be a participant in this research. There will be no other consent process or contact about the survey.

* Required

1. **Selecting "Agree" indicates that (1) you have read the above information, (2) you voluntarily agree to participate, (3) you are 18 years of age or older.** *
   Please select your choice below.
   Mark only one oval.

   ◯ Agree
   ◯ Disagree *Stop filling out this form.*

Background

2. **I am a:** *
Mark only one oval.

- ⬭ Undergraduate Student *After the last question in this section, skip to question 10.*
- ⬭ Research Scientist (Graduate Student, Primary Investigator, Post-doc, Research Assistant, Technician, etc) *After the last question in this section, skip to question 7.*
- ⬭ Adult in a non-science career *After the last question in this section, skip to question 10.*
- ⬭ Adult in a science-related career *After the last question in this section, skip to question 10.*

3. **My gender is:**

____

4. **I have received formal scientific education after high school (science classes in college, work in a lab, etc)** *
Mark only one oval.

- ⬭ Yes
- ⬭ No
- ⬭ Other: ____________________________

5. **Please say how much you prefer the following ways of getting new scientific information:** *
Phone users: Turn your phone sideways for easier viewing
Mark only one oval per row.

	Not at all	A bit	Average	Slightly Prefer	Highest Preference
Videos (TV, youtube, etc)	⬭	⬭	⬭	⬭	⬭
Infographics (Quick images of data often shared on facebook or twitter)	⬭	⬭	⬭	⬭	⬭
Reading the original research paper	⬭	⬭	⬭	⬭	⬭
Audio Sources (Radio, podcasts, etc)	⬭	⬭	⬭	⬭	⬭
Written summaries (news articles, wikipedia, blogs, etc)	⬭	⬭	⬭	⬭	⬭

6. **I enjoy learning about new scientific research** *
Mark only one oval.

	1	2	3	4	5	
Not at all	⬭	⬭	⬭	⬭	⬭	Yes definitely

Scientist Follow Up

7. **Please say how much you prefer the following ways of getting research updates OUTSIDE your field of study:** *

Mark only one oval per row.

	Not at all	A bit	Average	Slightly Prefer	Highest Preference
Recommendations from friends and colleagues					
Scientific Journals					
Social Media (twitter/instagram/facebook/etc)					
Newspaper Articles					

8. **Please say how much you prefer the following ways of getting research updates IN your field of study:** *

Mark only one oval per row.

	Not at all	A bit	Average	Slightly Prefer	Highest Preference
Pubmed or other alerts					
Social Media (twitter/instagram/facebook/etc)					
Newspaper Articles					
Scientific Journals					
Recommendations from friends and colleagues					

9. **I generally find research papers easy to understand** *

Mark only one oval.

	1	2	3	4	5	
Not at all						Yes definitely

Cohn et al. Research Summary

Please read the abstract below before moving to the next section.

This abstract is from: Cohn et al. “Clonal CD4+ T cells in the HIV-1 latent reservoir display a distinct gene profile upon reactivation” Nature Medicine 24, p604–609 (2018).

Despite suppressive combination antiretroviral therapy (ART), latent HIV-1 proviruses persist in patients. This latent reservoir is established within 48–72 h after infection, has a long half-life [1,2], enables viral rebound when ART is interrupted, and is the major barrier to a cure for HIV-1 [3]. Latent cells are exceedingly rare in blood (~1 per 1 × 106 CD4+ T cells) and are typically enumerated by indirect means, such as viral outgrowth assays [4,5]. We report a new strategy to purify and characterize single reactivated latent cells from HIV-1-infected individuals on suppressive ART. Surface expression of viral envelope protein was used to enrich reactivated latent T cells producing HIV RNA, and single-cell analysis was performed to identify intact virus. Reactivated latent cells produce full-length viruses that are identical to those found in viral outgrowth cultures and represent clones of in vivo expanded T cells, as determined by their T cell receptor sequence. Gene-expression analysis revealed that these cells share a transcriptional profile that includes expression of genes implicated in silencing the virus. We conclude that reactivated latent T cells isolated from blood can share a gene-expression program that allows for cell division without activation of the cell death pathways that are normally triggered by HIV-1 replication.

References:

1. Crooks, A. M. et al. Precise quantitation of the latent HIV-1 reservoir: implications for eradication

strategies. J. Infect. Dis. 212, 1361–1365 (2015).

2. Siliciano, J. D. et al. Long-term follow-up studies confirm the stability of the latent reservoir for HIV-1 in resting CD4+ T cells. Nat. Med. 9, 727–728 (2003).

3. Murray, A. J., Kwon, K. J., Farber, D. L. & Siliciano, R. F. The latent reservoir for HIV-1: how immunologic memory and clonal expansion contribute to hiv-1 persistence. J. Immunol. 197, 407–417 (2016).

4. Henrich, T. J., Deeks, S. G. & Pillai, S. K. Measuring the size of the latent human immunodeficiency virus reservoir: the present and future of evaluating eradication strategies. J. Infect. Dis. 215, S134–S141 (2017). suppl_3.

5. Spina, C. A. et al. An in-depth comparison of latent HIV-1 reactivation in multiple cell model systems and resting CD4+ T cells from aviremic patients. PLoS Pathog. 9, e1003834 (2013).

10. **I have read the abstract above.** *

Mark only one oval.

- ( ) Yes
- ( ) No

Cohn et al. Follow Up Questions

Do not return to the previous page.

11. **This research focuses on:** *

Mark only one oval.

- ( ) HIV
- ( ) FIV
- ( ) Influenza
- ( ) I don't know

12. **Check all the sentences about the research that are true** *

Check all that apply.

- [ ] Captured latent cells have higher expression of genes that increase virus activation
- [ ] Latent cells are a consequence of the lifecycle of the virus mentioned
- [ ] Latent cells captured from patient blood are mostly from a single latent cell that divided
- [ ] The capture technique is a type of cure for the virus discussed in the summary
- [ ] This research created a capture technique to collect all T-cells from patients

13. **Please rank the following statements:** *

Mark only one oval per row.

	Not at all	A bit	Average	Mostly	Very much
I enjoyed reading this abstract	( )	( )	( )	( )	( )
I understand this research more after reading this abstract	( )	( )	( )	( )	( )
I want to get more science updates via written abstract after reading this	( )	( )	( )	( )	( )

Takata et al. Research Summary

Please read the abstract below before moving to the next section.

This abstract is from: Takata et al. “CG dinucleotide suppression enables antiviral defence targeting non-self RNA” Nature 550, p124–127, (2017).

Vertebrate genomes exhibit marked CG suppression—that is, lower than expected numbers of 5′-CG-3′ dinucleotides [1]. This feature is likely to be due to C-to-T mutations that have accumulated over hundreds of millions of years, driven by CG-specific DNA methyl transferases and spontaneous methyl-cytosine deamination. Many RNA viruses of vertebrates that are not substrates for DNA methyl transferases mimic the CG suppression of their hosts [2,3,4]. This property of viral genomes is unexplained [4,5,6]. Here we show, using synonymous mutagenesis, that CG suppression is essential for HIV-1 replication. The deleterious effect of CG dinucleotides on HIV-1 replication was cumulative, associated with cytoplasmic RNA depletion, and was exerted by CG dinucleotides in both translated and non-translated exonic RNA sequences. A focused screen using small inhibitory RNAs revealed that zinc-finger antiviral protein (ZAP) [7] inhibited virion production by cells infected with CG-enriched HIV-1. Crucially, HIV-1 mutants containing segments whose CG content mimicked random nucleotide sequence were defective in unmanipulated cells, but replicated normally in ZAP-deficient cells. Crosslinking–immunoprecipitation–sequencing assays demonstrated that ZAP binds directly and selectively to RNA sequences containing CG dinucleotides. These findings suggest that ZAP exploits host CG suppression to identify non-self RNA. The dinucleotide composition of HIV-1, and perhaps other RNA viruses, appears to have adapted to evade this host defence.

References:

1. Karlin, S. & Mrázek, J. Compositional differences within and between eukaryotic genomes. Proc. Natl Acad. Sci. USA 94, 10227–10232 (1997)

2. Karlin, S., Doerfler, W. & Cardon, L. R. Why is CpG suppressed in the genomes of virtually all small eukaryotic viruses but not in those of large eukaryotic viruses? J. Virol. 68, 2889–2897 (1994)

3. Rima, B. K. & McFerran, N. V. Dinucleotide and stop codon frequencies in single-stranded RNA viruses. J. Gen. Virol. 78, 2859–2870 (1997)

4. Greenbaum, B. D., Levine, A. J., Bhanot, G. & Rabadan, R. Patterns of evolution and host gene mimicry in influenza and other RNA viruses. PLoS Pathog. 4, e1000079 (2008)

5. Cheng, X. et al. CpG usage in RNA viruses: data and hypotheses. PLoS One 8, e74109 (2013)

6. Futcher, B. et al. Reply to Simmonds et al.: Codon pair and dinucleotide bias have not been functionally distinguished. Proc. Natl Acad. Sci. USA 112, E3635–E3636 (2015)

7. Gao, G., Guo, X. & Goff, S. P. Inhibition of retroviral RNA production by ZAP, a CCCH-type zinc finger protein. Science 297, 1703–1706 (2002)

14. **I have read the abstract above.** *

Mark only one oval.

- Yes
- No

Takata et al. Follow Up Questions

Do not return to the previous page.

15. **This research focuses on:** *

Mark only one oval.

- HIV
- FIV
- Influenza
- I don't know

16. **Check all the sentences about the research that are true** *

Check all that apply.

- Vertebrates have evolved less AG nucleotide pairs
- ZAP interacts with the DNA of the virus mentioned in the summary
- ZAP is a protein that is made by the infected host cell
- The virus mentioned has evolved to lack CG pairs to avoid cell anti-viral defenses
- All possible DNA nucleotide pairs show up at the same rate as each other in vertebrates (eg. AT is present at the same frequency as GT or CG or GC)

17. **Please rank the following statements:** *

Mark only one oval per row.

	Not at all	A bit	Average	Mostly	Very much
I enjoyed reading this abstract					
I understand this research more after reading this abstract					
I want to get more science updates via written abstract after reading this					

18. **Comments:**

Powered by
Google Forms
