# Supplementary material for: Video abstracts and plain language summaries are more effective than graphical abstracts and published abstracts
Source: PLoS One. 2019 Nov 19;14(11):e0224697. doi: 10.1371/journal.pone.0224697 (PMC6863540; doi:10.1371/journal.pone.0224697)
Supplement: Attachment — Submitted filename: REVIEW 2 PLOS.docx [file pone.0224697.s008.docx]

This manuscript has a wide range of information and is a little bit difficult to follow. The changes made in the first review solved many of these problems and made it really easier and organized. That said, I noted a few more things that could be adjusted in this version once I could get a better idea of the message you are trying to send. Here are my considerations:

1. If you go to Cochrane Methods you can find a section on Methodological Expectations of Cochrane Intervention Reviews (MECIR) (<https://methods.cochrane.org/methodological-expectations-cochrane-intervention-reviews>). There you will find a link to the Standards for the reporting of plain language summaries of new reviews of interventions by the Plain Language Expectations for Authors of Cochrane Summaries (PLEACS Initiative). (<https://methods.cochrane.org/sites/default/files/public/uploads/pleacs_2019.pdf>)

2. Lines 130-7 of introduction seems like methods to me. If you could take this part then the paragraph you be as follows, that way you would say what and why you did on your research.

*“To evaluate the effectiveness of different summary types for people with different careers, we created a survey that presents participants with a video abstract, graphical abstract, plain language summary, or published abstract from two papers in the same subject area. The survey looked at comprehension, perceived understanding, enjoyment, and whether the participants wanted to see more summaries of that type (…)”*

3. Line 238: *“input their gender if they so desired (a fill-in-the-blank that was not required)”*

Were there many people who didn’t fill this? Once this was something that you reported I think this topic should have been mandatory, but that is not something you can change at this point. I Believe, then, that this (the number of people that didn’t answer this topic) should be reported when you inform the proportion of men/women that answered the survey.

4. Line 367: *“Given the clear reported preference for written forms of communication (fig 2A), it was expected that the plain language summaries and perhaps the published abstracts would be the most effective summaries when tested (…) but videos were either as effective or more effective than plain language summaries in all cases except comprehension of science related participants for the Cohn et al. paper where plain language summaries had a higher average score (M=4 of 6 for video, M=5 of 6 for plain language) (fig 3)”*

I believe this deserves some kind of discussion. Although the most preferable format is the written one, the videos were the easier format for comprehension. How would the authors explain that? A recently published paper from physiotherapy journal (<https://doi.org/10.1016/j.physio.2018.11.003>) analysed all the plain language summaries from the Physiotherapy Evidence Database and it found that only 2% of reports were considered at a suitable reading level for non scientific population! That could explain why the scientific community in addition to prefer, comprehend the message better than the rest of the people surveyed.

5. I understand the authors say they found a significant result on the correlation analysis and for that found it important to report, however, as the authors themselves report on the text, the correlation could have happened because one of the papers is harder to read once it requires more background knowledge to understand. Therefore, although significant this should be interpreted carefully as this may not reflect the true situation, mainly because these sort of resources usually target people that are not part of the scientific community or people from other areas of research, which, as consequence, need an easier text to understand the results of a very specific scientific study.

Also you could not analyse the correlation for plain language summaries, perhaps it would not be correlated since almost everyone preferred PLS but not all comprehend them. Again, if the plain language summaries were really easier to read (which they appear they are not) the preferred format could be the best for understanding (-but that is just a theory!)
